# Suplementação de Vitamina D

**DOI:** 10.36660/abc.20210181

**Published:** 2021-05-06

**Affiliations:** 1 UNESP Faculdade de Medicina de Botucatu Departamento de Clínica Médica BotucatuSP Brasil Departamento de Clínica Médica - Faculdade de Medicina de Botucatu (UNESP), Botucatu, SP - Brasil.; 2 Unimed Sorocaba São PauloSP Brasil Unimed Sorocaba, São Paulo, SP - Brasil.; 3 Universidade Federal de Mato Grosso do Sul Instituto Integrado de Saúde Campo GrandeMS Brasil Instituto Integrado de Saúde, Universidade Federal de Mato Grosso do Sul (UFMS), Campo Grande, MS - Brasil.

**Keywords:** Vitamina D, Estado Nutricional, Osso e Ossos/metabolismo, Suplementos Nutricionais/efeitos adversos

A vitamina D (Vit D) é uma vitamina lipossolúvel essencial para o metabolismo de ossos e minerais. O status de Vit D é avaliado medindo-se os níveis séricos de 25-hidroxivitamina D [25(OH)D]. Atualmente, a suplementação da Vit D é indicada principalmente em casos de deficiência. No entanto, existem duas questões importantes em relação à suplementação da Vit D. A primeira está relacionada à definição do limite inferior para os valores considerados normais de [25(OH)D] sérico. Nos últimos anos, extensa pesquisa clínica tem revelado que grandes proporções das populações globais têm baixos níveis de Vit D, isto é, concentrações séricas de [25(OH)D] menor que 20 ng/mL.^1^ Contudo, muitos pesquisadores consideram esse valor superestimado, considerando, assim, que um maior número de pessoas necessitaria de suplementação. Hoje, várias sociedades médicas estão em grande discussão sobre quando rastrear a deficiência de Vit D e quando suplementá-la.^1^^,^^2^

Outra questão a respeito da suplementação da Vit D diz respeito ao fato de que estudos experimentais e epidemiológicos convincentes sugerem que a deficiência da Vit D esteja associada ao maior risco de doenças cardiovasculares e doenças imunes crônicas, e câncer. Consequentemente, a suplementação da Vit D tem sido realizada na população geral sem uma indicação específica. Contudo, estudos mais recentes têm relatado que a suplementação da Vit D para a prevenção ou controle de doenças crônicas como o câncer, diabetes mellitus, demência e doenças cardiovasculares não tem mostrado benefícios.^3^^–^^5^ Além da ausência de benefícios, um estudo mostrou que a suplementação diária de Vit D foi associada a uma maior necessidade de dispositivos de suporte mecânico circulatório na insuficiência cardíaca avançada, o que indica que é necessário cautela em relação à suplementação em longo prazo.^6^

Estudos experimentais são importantes, pois permitem estabelecer melhor controle de parâmetros envolvidos na suplementação da vitamina.^7^^–^^9^ Na edição atual dos ABC, Santos et al.^10^ confirmaram sua hipótese de que a suplementação com Vit D em doses que não levam à hipercalcemia (doses “não hipercalcêmicas”) induz mudanças deletérias no miocárdio em ratos, e que esse processo pode, ao menos em parte, ser modulado pela proteína de interação com a tiorredoxina (TXNIP), pela tiorredoxina (Trx), e pelo estresse oxidativo. Em um elegante estudo, ratos Wistar machos foram submetidos a duas diferentes doses não hipercalcêmicas por dois meses. A suplementação reduziu a atividade das enzimas envolvidas no metabolismo oxidativo e aumentou a via glicolítica. O estresse oxidativo foi caracterizado por peroxidação lipídica aumentada e atividade reduzida das enzimas antioxidantes no miocárdio dos ratos suplementados. Além disso, uma maior expressão da TXNIP e menor atividade da Trx, associadas com redução nos marcadores antiapoptóticos, foram observados na suplementação com a maior dose de Vit D, de maneira dose-dependente. Considerando o aumento no estresse oxidativo e a diminuição nos marcadores antiapoptóticos, podemos levantar a hipótese de que, em longo prazo, as alterações no miocárdio poderiam induzir a remodelação cardíaca ou predispor corações sadios a efeitos deletérios da lesão cardíaca, tais como isquemia miocárdica e hipertensão arterial. Conforme destacada pelos autores, uma limitação do estudo é o curto período de tratamento, o que não permitiu determinar se a suplementação crônica de Vit D causa remodelação cardíaca patológica.

Apesar de dados epidemiológicas associarem a Vit D a desfechos cardiovasculares, e corroborarem um papel da Vit D no processo patogênico, dados mecanicistas são ainda insuficientes para se recomendar a suplementação da vitamina para prevenção ou tratamento de outras doenças que não a doença óssea metabólica.^11^
