# Supplementary material for: Suplementação de Vitamina D
Source: Arq Bras Cardiol. 2021 May 6;116(5):979–80. [Article in Portuguese] doi: 10.36660/abc.20210181 (PMC8121470; doi:10.36660/abc.20210181)
Supplement: Translation

# Translation: Supplementation of Vitamin D

**Affiliations:** 1 BotucatuSP Brazil Departamento de Clínica Médica - Faculdade de Medicina de Botucatu (UNESP), Botucatu, SP - Brazil.; 2 São PauloSP Brazil Unimed Sorocaba, São Paulo, SP - Brazil.; 3 Campo GrandeMS Brazil Instituto Integrado de Saúde, Universidade Federal de Mato Grosso do Sul (UFMS), Campo Grande, MS - Brazil.

**Keywords:** Vitamin D, Nutritional Status, Bone and Bones/metabolism, Dietary Supplements/adverse effects

Vitamin D (Vit D) is a fat-soluble vitamin that is essential in mineral and bone metabolism. Vit D status is evaluated by measuring serum 25-hydroxyvitamin D [25(OH)D]) levels. Currently, Vit D supplementation is mainly indicated in cases of vitamin deficiency. However, there are two main issues concerning Vit D supplementation. The first is related to the definition of the lower limit of normal for serum 25(OH) D. In recent years, extensive clinical research has revealed that large percentages of global populations have low Vit D levels, i.e., serum [25(OH)D] concentrations below 20 ng/mL.^1^ However, several investigators have considered that this value is probably overestimated, therefore putting more people in need for supplementation. Several medical societies are now intensively debating on when to screen for Vit D deficiency and when to supplement Vit D.^1^^,^^2^

The other issue concerning Vit D supplementation relates to the fact that convincing experimental and epidemiological studies have suggested that Vit D deficiency is associated with increased risk of chronic cardiovascular and immunological diseases and cancer. Therefore, Vit D has been supplemented in the general population without a specific indication. However, more recent studies have reported that Vit D supplementation for preventing or controlling chronic diseases such as cancer, diabetes mellitus, dementia or cardiovascular disease has failed to provide benefits.^3^^–^^5^ Furthermore, not only were no benefits found. In advanced heart failure, a daily Vit D supplement was associated with a greater need for mechanical circulatory support devices, which indicates caution regarding long-term supplementation.^6^

Experimental studies are important as they allow the establishment of better control parameters involved in vitamin supplementation.^7^^–^^9^ In the current issue of ABC, Santos et al.^10^ confirmed their hypotheses that Vit D supplementation at non-hypercalcemic doses induces detrimental myocardial changes in rats and that this process may, at least in part, be modulated by thioredoxin-interacting protein (TXNIP), thioredoxin (Trx), and oxidative stress. In an elegant study, male Wistar rats were subjected to two different non-hypercalcemic Vit D doses for two months. Supplementation decreased the activity of enzymes involved in oxidative metabolism and increased the glycolytic pathway. Increased oxidative stress was characterized by higher lipid peroxidation and reduced antioxidant enzyme activity in myocardium of the supplemented rats. Additionally, higher TXNIP expression and lower Trx activity, associated with reduced antiapoptotic markers, were also observed with the higher dose of Vit D, in a dose-dependent manner. Considering the increased oxidative stress and reduced antiapoptotic markers, we can hypothesize that in the long-term the myocardial changes could induce cardiac remodeling or predispose healthy hearts to deleterious effects of cardiac injury, such as myocardial ischemia and arterial hypertension. As pointed out by the authors, one limitation of the study is the short treatment period, which did not allow to determine whether chronic Vit D supplementation causes pathological cardiac remodeling.

Although epidemiological data link Vit D to cardiovascular outcomes and support a role for Vit D in pathogenic processes, mechanistic data are insufficient to recommend Vit D supplementation for prevention or treatment of diseases other than bone metabolic disease.^11^
